# Consumption of ultra-processed foods and health outcomes: a systematic review of epidemiological studies

**DOI:** 10.1186/s12937-020-00604-1

**Published:** 2020-08-20

**Authors:** Xiaojia Chen, Zhang Zhang, Huijie Yang, Peishan Qiu, Haizhou Wang, Fan Wang, Qiu Zhao, Jun Fang, Jiayan Nie

**Affiliations:** 1 Department of Gastroenterology, Zhongnan Hospital of Wuhan University, No. 169, Donghu Road, Wuchang District, Wuhan, 430071 Hubei Province China; 2 Hubei Clinical Center & Key Lab of Intestinal & Colorectal Diseases, Wuhan, China

**Keywords:** Ultra-processed foods, Noncommunicable diseases, Health, Systematic review

## Abstract

**Background:**

Consumption of ultra-processed foods (UPFs) plays a potential role in the development of obesity and other diet-related noncommunicable diseases (NCDs), but no studies have systematically focused on this. This study aimed to summarize the evidence for the association between UPFs consumption and health outcomes.

**Methods:**

A comprehensive search was conducted in PubMed, Embase, and Web of Science to identify all relevant studies. Epidemiological studies were included, and identified studies were evaluated for risk of bias.A narrative review of the synthesized findings was provided to assess the association between UPFs consumption and health outcomes.

**Results:**

20 studies (12 cohort and 8 cross-sectional studies) were included in the analysis, with a total of 334,114 participants and 10 health outcomes. In a narrative review, high UPFs consumption was obviously associated with an increased risk of all-cause mortality, overall cardiovascular diseases, coronary heart diseases, cerebrovascular diseases, hypertension, metabolic syndrome, overweight and obesity, depression, irritable bowel syndrome, overall cancer, postmenopausal breast cancer, gestational obesity, adolescent asthma and wheezing, and frailty. It showed no significant association with cardiovascular disease mortality, prostate and colorectal cancers, gestational diabetes mellitus and gestational overweight.

**Conclusions:**

This study indicated a positive association between UPFs consumption and risk of several health outcomes. Large-scale prospective designed studies are needed to confirm our findings.

## Introduction

Noncommunicable diseases (NCDs), such as cardiovascular diseases, type 2 diabetes and some cancers, are collectively responsible for almost 70% of all deaths worldwide. The current prevalence of NCDs poses devastating health outcomes and constitutes a serious threat to global health systems. To reduce the number of deaths caused by NCDs, a better understanding of the potential risk factors is needed.

Unhealthy diets are recognized as a major determinant of the occurrence of NCDs. With the increasing trend of NCDs, a steady rise in the share of processing foods has been seen. In the last half century food processing has evolved greatly as a consequence of the industrialization and globalization of food systems [1]. Negative effects on nutritional dietary quality emerged subsequently, such as higher content in free sugars, saturated fats, energy density and sodium, and less content in protein, fiber and micronutrients. It is believed that most NCDs can be prevented by changes in diet patterns.

Ultra-processed foods (UPFs) are defined as formulations of ingredients derived from foods and additives, coupled with substances including colorings, flavorings, sweeteners, and emulsifiers [2]. They contain little if any intact food. Included in this definition are sugar-sweetened beverages, sweets, ice cream, chocolates, savoury snacks, burgers, processed meat and frozen dishes. Compared with other food groups, UPFs are typically durable, ready to consume, low-cost and hyper-palatable. They tend to be packaged delicately and marketed concentratedly. They are characteristically fatty, sugary or salty, energy-dense and lack of protein, dietary fibre, micronutrients and several bioactive compounds [3–5]. Furthermore, they may contain neo-formed contaminants derived from industrial processing, as well as substances from additives and packaging [6, 7]. Considering the association between UPFs and poorer dietary quality, the share of UPFs has been proposed as an effective predictor of population diet quality [8–10].

The whole world has witnessed a dramatic transition in food consumption patterns. Unprocessed or minimally processed foods and freshly prepared meals are gradually displaced by UPFs. The shift appeared initially in high and middle income countries, and then worldwide [11, 12]. Transnational corporations are major factors that drive the production and sales of UPFs, along with their convenience, branding and aggressive marketing [13]. These characteristics create massive market advantages for UPFs over other food groups [14]. In high income countries, more than half of the foods consumed are UPFs for most of the age groups, and consumption decreases with age [15, 16]. Purchase surveys and dietary trends on UPFs consumption have been performed in Asia and many western countries [17–21]. It has been evaluated that the energy contribution of UPFs ranged from 25 to 60% [22].

The existing evidence indicates that displacement by UPFs is driving a rising prevalence of obesity and other diet-related NCDs [23]. A growing body of evidence suggests that increases of UPFs in dietary proportion were associated with a higher incidence of adverse health outcomes [16, 24–42]. Decreasing the dietary share of UPFs may notably contribute to the prevention of diet-related NCDs [43–45].

As UPFs are increasing dominantly during the past decades, understanding their potential impacts on health outcomes has become a major imperative. To date, however, this literature has not been comprehensively evaluated. No reviews have been conducted on this topic previously. To address these concerns, this systematic review was conducted to summarize the evidence for the association between UPFs consumption and health outcomes.

## Methods

### Study design

This systematic review is completed according to the MOOSE (Meta-analysis Of Observational Studies in Epidemiology) Statement [46]. We developed a protocol with methods of the review in advance (Supplement 1).

### Search strategy

The public databases of PubMed, Embase, and Web of Science were comprehensively searched for relevant studies published up to October 11, 2019. Broad search strategy was used to ensure that no publications were overlooked. The search terms were listed in Supplement 1. Studies in language other than English were excluded. Reference lists of relevant articles and some key journals were also hand-searched for other pertinent studies. We considered no limitations on the publication date.

### Eligibility criteria

Epidemiological studies including cohort and cross-sectional studies were considered for further screening. Eligibility was assessed independently by two authors (Xiaojia Chen and Zhang Zhang). All differences were resolved by consensus with a third author (Fan Wang). We included studies meeting the following inclusion criteria: (i) included more than 500 participants; (ii) the exposure of interest was consumption of UPFs and the outcomes of interest were any health outcomes (e.g., all-cause mortality, cancers); (iii) reported the effect sizes of hazard ratios (HRs), odds ratios (ORs) or relative risks (RRs) with 95% confidence intervals (CIs). We excluded experimental studies, reviews, letters, editorials, and abstracts without full texts. When more than one studies reported on the same cohort and outcome, we only included the study with the longest follow-up.

### Data extraction

Both authors independently reviewed full-texts of the eligible studies and extracted data using a standardized collection form. All differences were resolved by consensus. Information extracted from each study was as follows: first author, year of publication, study design, study period and area, study population, number of participants, exposure assessment, outcome measures and categories, comparison, effect sizes (HRs, ORs, and RRs) with 95% CIs. All kinds of measures of exposures and outcomes were allowed. There was also no limitation on outcome categories.

### Quality assessment

Risk of bias was evaluated by two reviewers independently. All differences were resolved by consensus. We assessed the quality of cohort studies with the Newcastle-Ottawa Scale (NOS) and cross-sectional studies with the JBI Critical Appraisal Checklist [47, 48]. Risk of bias of each eligible study was evaluated according to a series of methodological features: (i) sampling of participants and their representativeness of the population; (ii) assessment of exposure to UPFs; (iii) ascertainment of health outcomes; (iv) adjustment for potential confounders; (v) demonstration was mentioned that outcome of interest was not present at start of study. In general, cohort studies scoring ≥6 were considered as high quality, while cross-sectional studies with ≥5 “yes” were rated as high quality.

### Data synthesis

In light of the overall low number of studies, variance of exposures and outcomes measurement, no quantitative meta-analysis was conducted. To systematically synthesize findings across included studies, a narrative synthesis approach was chosen. We tabulated study characteristics and classified studies into groups according to different health outcomes. The evidence was synthesized to provide useful insights for the association of interest.

## Results

### Study selection and characteristics

**Fig. 1:**
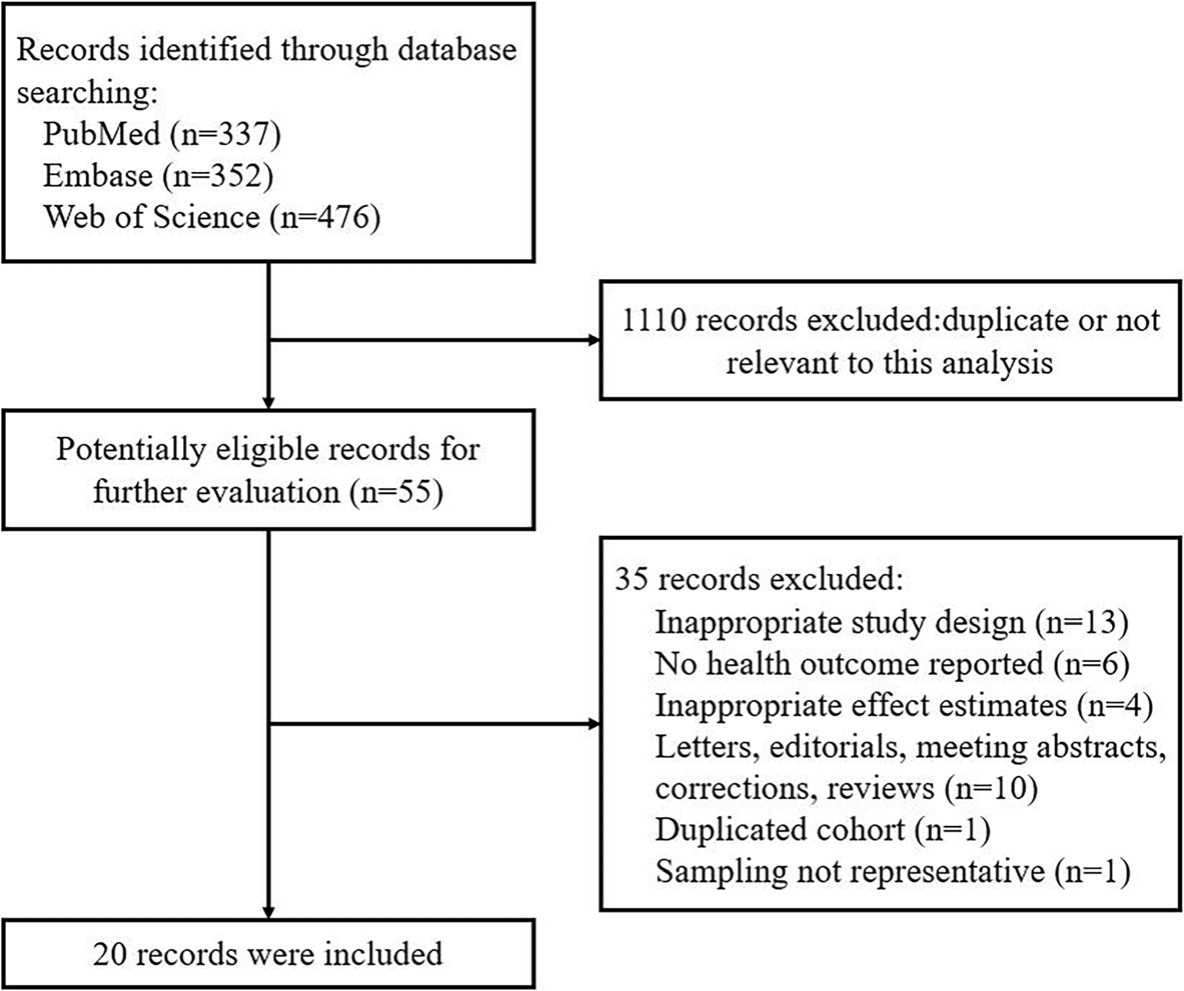
Flowchart of literature search

The search strategy identified 1165 records. 563 articles were screened by titles and abstracts after duplicates removed. Of the 55 full-texts assessed for eligibility, 20 published epidemiological studies (12 cohort and 8 cross-sectional studies) were included into the systematic review, with a total of 334,114 participants and 10 diseases (Fig. 1). An overview of the characteristics of included studies was provided in Table 1. All studies were published between 2015 and 2019, with a sample size ranging from 785 to 109,104. Six studies were conducted in Spain, while 5 in France, 4 in Canada, 3 in America and 2 in Brazil. The median follow-up ranged from 3.5 to 19 years in cohort studies. The mean age of participants was between 28 and 69 years, exclusive of the unknown one [41]. The female proportion ranged from 49 to 100%. Of the 20 eligible studies, 4 focused on all-cause mortality [24–27], 2 on cardiocerebrovascular diseases [28, 29], 2 on metabolic syndrome [30, 31], 5 on overweight and obesity [16, 32–35], 2 on mental health diseases [36, 37]. The remaining 5 studies respectively investigated gastrointestinal diseases [38], cancers [39], pregnancy outcome [40], respiratory diseases [41], and geriatric diseases [42].

**Table 1:** Characteristics of included studies

First author, year	Study design	Study period, area	Study population, age	Sample size	Exposure measures	Health outcome	Outcome measures	Comparison	Effect size (95% CI)
Rico-Campà, 2019 [24]	Prospective cohort study	1999–2014, Spain	University graduates, 20-91y	19,899	FFQ	All-cause mortality	Medical records, database	Q4 vs. Q1	HR: 1.62 (1.13, 2.33)
Schnabel, 2019 [25]	Prospective cohort study	2009–2017, France	Adults participants, ≥45y	44,551	Dietary records, interview, biomarkers	All-cause mortality	Clinical data	Q4 vs. Q1	HR: 1.25 (0.99, 1.57)
Blanco-Rojo, 2019 [26]	Prospective cohort study	2008–2016, Spain	Adult participants, ≥18y	11,898	Questionnaire	All-cause mortality	Computerized search	Q4 vs. Q1	HR: 1.44 (1.01, 2.07)
Kim, 2019 [27]	Prospective cohort study	1988–2011, USA	Adult participants, ≥20y	11,898	Dietary records, questionnaire	All-cause mortality Cardiovascular disease mortality	Database	Q4 vs. Q1	HR: 1.30 (1.08, 1.57) HR: 1.13 (0.74, 1.71)
Srour, 2019 [28]	Prospective cohort study	2009–2018, France	Adult participants, ≥18y	105,159	Dietary records, interview, biomarkers	All cardiovascular diseases Coronary heart diseases Cerebrovascular diseases	Questionnaire, medical records, database	Q4 vs. Q1	HR: 1.23 (1.04, 1.45) HR: 1.18 (0.93, 1.52) HR: 1.23 (1.00, 1.53)
Mendonça, 2017 [29]	Prospective cohort study	1999–2015, Spain	University graduates, 20-91y	14,790	FFQ	Hypertension	Self-reported, clinical data	T3 vs. T1	HR: 1.21 (1.06, 1.37)
Melo, 2018 [41]	Cross-sectional study	2012, Brazil	9th graders, NC	109,104	Questionnaire	Asthma Wheezing	Questionnaire	Q5 vs. Q1	OR: 1.27 (1.15, 1.41) OR: 1.42 (1.35, 1.50)
Schnabel, 2018 [38]	Prospective cohort study	2008–2018, France	Adult participants, ≥18y	33,343	Dietary records, questionnaire	Functional gastrointestinal disorders	Questionnaire, medical history, symptoms	Q4 vs. Q1	OR: 1.25 (1.12, 1.39)
Adjibade, 2019 [36]	Prospective cohort study	2009–2018, France	Adult volunteers, ≥18y	26,730	Dietary records	Depression	Clinical data	Q4 vs. Q1	HR: 1.30 (1.15, 1.47)
Gómez-Donoso, 2018 [37]	Prospective cohort study	1999–2016, Spain	University graduates, NC	14,907	FFQ	Depression	Questionnaire, clinical interview	Q4 vs. Q1	HR: 1.33 (1.07, 1.64)
Steele, 2019 [30]	Cross-sectional study	2009–2014, USA	Adult participants, ≥20y	6385	Interview	Metabolic syndrome	Interviews, health examination	Q5 vs. Q1	OR: 1.28 (1.09, 1.50)
Lavigne-Robichaud, 2018 [31]	Cross-sectional study	2005–2009, Canada	Adult participants, ≥18y	811	Dietary records	Metabolic syndrome	Clinical data	Q5 vs. Q1	OR: 1.90 (1.14, 3.17)
Juul, 2018 [32]	Cross-sectional study	2005–2014, USA	Adults participants, 20–64y	15,977	Interview	Overweight Obesity Abdominal obesity	Clinical data	Q5 vs. Q1	OR: 1.48 (1.25, 1.76) OR: 1.53 (1.29, 1.81) OR: 1.62 (1.39, 1.89)
Louzada, 2015 [33]	Cross-sectional study	2008–2009, Brazil	Individuals, ≥10y	30,243	Dietary records	Overweight Obesity	Clinical data	Q5 vs. Q1	OR: 1.26 (0.95, 1.69) OR: 1.98 (1.23, 3.12)
Mendonca, 2016 [35]	Prospective cohort study	1999–2012, Spain	University graduates, middle-aged	8451	FFQ	Overweight	Self-reported	Q4 vs. Q1	HR: 1.26 (1.10, 1.45)
Nardocci, 2019 [16]	Cross-sectional study	2004–2005, Canada	Adult participants, ≥18y	19,363	Dietary records	Obesity	Clinical data (32% self-reported)	Q5 vs. Q1	OR: 1.32 (1.05, 1.57)
Silva, 2018 [34]	Cross-sectional study	2008–2010, Brazil	Civil servants from universities and research organizations, 35–64y	8977	FFQ	Overweight Obesity	Clinical data	Q4 vs. Q1	OR: 1.31 (1.13, 1.51) OR: 1.41 (1.18, 1.69)
Fiolet, 2018 [39]	Prospective cohort study	2009–2017, France	Adult volunteers, ≥18y	104,980	Dietary records	Overall cancer Prostate cancer Colorectal cancer Breast cancer	Questionnaire, medical records, database	Q4 vs. Q1	HR: 1.23 (1.08, 1.40) HR: 0.93 (0.61, 1.40) HR: 1.23 (1.08, 1.40) HR: 1.13 (0.89, 1.42)
Sartorelli, 2019 [40]	Cross-sectional study	2011–2012, Brazil.	Adult women, ≥20y	785	Dietary records	Obesity Overweight Gestational diabetes mellitus	Clinical data	T3 vs. T1	OR: 3.06 (1.27, 3.37) OR: 1.17 (0.75, 1.82) OR: 0.82 (0.49, 1.36)
Sandoval-Insausti, 2019 [42]	Prospective cohort study	2008–2012, Spain	Individuals, ≥60y	1822	Interview	Frailty	Clinical data	Q4 vs. Q1	OR: 3.67 (2.00, 6.73)

*Abbreviation*: *FFQ* food frequency questionnaire, *HR* hazard ratio, *OR* odds ratio, *Q* quartile or quintile, *T* tertile, *NC* not clear

### Quality assessment

The quality assessment was listed in Supplement 2. Cohort studies scored ranging from 6 to 9. A maximum of 9 points could be awarded to each cohort study: 4 for selection, 2 for comparability, and 3 for outcome. Two cohort studies represented the highest quality. The most common bias risk was incomplete representativeness. Volunteers [25, 28, 36, 38, 39] and university graduates [29, 35, 37] tended to be more health-conscious and had healthier dietary habits, leading to a lack of representativeness of the general population. Considering the chronic development of NCDs, the insufficient follow-up was another source of bias risk [39, 42].

Cross-sectional studies achieved 5 to 8 “yes”. Three studies were unclear whether the measurement of the condition was assessed according to the objective and standard criteria [30, 31, 41]. Three studies applied incomplete statistical analysis [31, 34, 40]. The methods of exposures [33, 40] or outcomes [15, 41] measurement were short of validity and reliability, which led to bias risk. However, all the eligible studies made adequate adjustments for potential confounding factors. Generally, all included studies had a good methodological quality.

### Study results

The results were synthesized in Table 1, comprising study design, study setting, samples, exposures, outcomes, and effect sizes. A narrative synthesis of our findings is as follows.

### All-cause mortality

Four cohort studies investigated the association between UPFs consumption and risk of all-cause mortality [24–27]. Despite diverse methods of exposures assessment, all studies reported a significant positive association, indicating that high consumption of UPFs was associated with an increased hazard for all-cause mortality. Three of them conducted sensitivity analyses and results did not substantially change, showing the strength of the association [24, 25, 27]. Rico-Campà et al. and Schnabel et al. found that cancer was the main cause of death. However, Kim et al. reported null association with cardiovascular disease mortality, which is surprising.

### Cardiocerebrovascular diseases

Two cohort studies investigated the association between UPFs consumption and risk of cardiocerebrovascular diseases [28, 29]. Srour et al. focused on the overall of cardiovascular diseases, and Mendonca et al. on hypertension. Even after adjustment for potential confounding factors, it was found that high UPFs consumption increased the risk of overall cardiovascular diseases (HR: 1.23, 95% CI: 1.04 to 1.45), coronary heart diseases risk (HR: 1.18, 95% CI: 0.93 to 1.52), cerebrovascular diseases risk (HR: 1.23, 95% CI: 1.00 to 1.53), and hypertension (HR: 1.21, 95% CI: 1.06 to 1.37). Results from sensitivity analyses did not substantially change.

### Respiratory diseases

One cross-sectional study investigated the association between UPFs consumption and risk of asthma and wheezing among the Brazilian adolescents [41]. It found a positive association between UPFs consumption and risk of asthma (OR: 1.27, 95% CI: 1.15 to 1.41) and wheezing (OR: 1.42, 95% CI: 1.35 to 1.50). In addition, the direct association was stronger among male adolescents, those who did not consume fruits and vegetables regularly, non-smokers, with parents who did not smoke, and those living in non-capital cities.

### Gastrointestinal diseases

One cohort study investigated the association between UPFs consumption and risk of functional gastrointestinal disorders [38]. In a sample of French adults, it was found that high UPFs consumption increased the risk of irritable bowel syndrome (IBS) (OR: 1.25, 95% CI: 1.12 to 1.39) and concomitant functional dyspepsia (OR: 1.25, 95% CI: 1.05 to 1.47). No associations were observed between UPFs consumption and functional dyspepsia alone without concomitant IBS, indicating the indispensable role of IBS in the positive association.

### Mental health diseases

Two cohort studies investigated the association between UPFs consumption and risk of depression [36, 37]. Both reported a positive association even after extensive adjustment. The fourth quartile had a significantly increased risk compared to the lowest quartile. Similar results were observed after sensitivity analyses, confirming the robustness of the association.

### Metabolic syndrome

Two cross-sectional studies investigated the association between UPFs consumption and risk of metabolic syndrome [30, 31]. Both reported a significant positive association, suggesting the growing evidence of associations between UPFs consumption and several diet-related NCDs. In addition, Steele et al. observed that the association was stronger among young adults and decreased with age.

### Overweight and obesity

Four cross-sectional studies [16, 32–34] and one prospective cohort study [35] investigated the association between UPFs consumption and risk of overweight (BMI ≥ 25 kg/m^2^) and obesity (BMI ≥ 30 kg/m^2^). Three studies reported a positive association for overweight [32, 33, 35] and four studies for obesity [16, 32–34]. Furthermore, Juul et al. found a positive association between high UPFs consumption and abdominal obesity (OR: 1.62, 95% CI: 1.39 to 1.89). Stronger effects were observed among women, partly due to sex-related differences in food choices [32, 33]. The evidence strongly supported the role of increased UPFs consumption in the obesity epidemic worldwide.

### Cancers

One cohort study investigated the association between UPFs consumption and risk of cancers [39]. After a relatively short median follow-up of 5 years, this volunteer-based study suggested a positive association between UPFs consumption and overall cancer risk (HR: 1.23, 95% CI: 1.08 to 1.40) and postmenopausal breast cancer risk (HR: 1.38, 95% CI: 1.05 to 1.81). No significant association was observed for prostate, colorectal, overall breast and premenopausal breast cancers. However, a direct association about overall breast cancer risk was obtained when UPFs consumption was regarded as a continuous variable.

### Pregnancy outcome

One cross-sectional study investigated the association between UPFs consumption and risk of several pregnancy outcomes [40]. This study was conducted among adult women with singleton pregnancies. It detected a positive association between UPFs consumption and pregnant obesity (OR: 3.06, 95% CI: 1.27 to 3.37), but no significant association was observed in gestational diabetes mellitus (OR: 0.82, 95% CI: 0.49 to 1.36) and overweight (OR: 1.17, 95% CI: 0.75 to 1.82).

### Geriatric diseases

One cohort study investigated the association between UPFs consumption and risk of incident frailty in the old adults [42]. With a relatively short median follow-up of 3.5 years, this study suggested a positive association between UPFs consumption and frailty risk (OR: 3.67, 95% CI: 2.00 to 6.73). Similar results were observed in sensitivity analyses.

## Discussion

To the best of our knowledge, this is the first systematic review of available epidemiological evidence on the association between UPFs consumption and health outcomes. We identified 12 cohort and 8 cross-sectional studies, and found that there was a positive association between UPFs consumption and risk of all-cause mortality, overall cardiovascular diseases, coronary heart diseases, cerebrovascular diseases, hypertension, metabolic syndrome, overweight and obesity, depression, irritable bowel syndrome, overall cancer, postmenopausal breast cancer, gestational obesity, adolescent asthma and wheezing, and frailty. It showed no obvious association with cardiovascular disease mortality, prostate and colorectal cancer, gestational diabetes mellitus and gestational overweight.

Among the included studies, different methods were applied to estimate intake of UPFs. In some studies portion sizes were estimated using validated photographs. They calculated energy and weight relative to total food intake according to specific food composition databases. Some studies calculated daily intake by multiplying the portion size by the frequency of consumption, which had been proved validated. A majority of included studies evaluated intake as the percentage to total energy while the others selected weight proportion. The application of energy proportion contributed to reduction of variation due to body size, metabolic efficiency, and physical activity [33]. Weight proportion was taken into account for UPFs that did not provide any energy intake such as artificially sweetened drinks, and factors related to food processing [38]. Although methods vary, UPFs intake was all modeled as quantiles (e.g., tertiles, quartiles and quintiles). We selected effect estimates adjusted by the most factors for the highest versus the lowest consumption levels, which made them comparable. In light of the increasing concern of UPFs, further research is needed to set standard methods for UPFs intake estimation.

Despite different ways to estimate intake of UPFs, all the studies conducted food classification using the NOVA system, except one [33]. Louzada et al. divided foods into three groups according to the degree of processing, which was also consistent with the NOVA system. NOVA, a food classification system which classifies foods into four groups according to the nature, extent and purpose of industrial processing, has now been applied globally [49]. Groups are as follows: (i) Unprocessed or minimally processed foods; (ii) Processed culinary ingredients; (iii) Processed foods; (iv) Ultra-processed foods. Instead of focusing on nutrient composition of the diet, it takes into consideration all physical, chemical and biological methods used during the food productive process [50]. The NOVA system has been used to describe population dietary patterns, assess changes in the dietary share of UPFs, and analyze the association of the dietary share with nutrient profile and with health outcomes [50]. It will contribute to the prevention of NCDs and the improvement of public health worldwide [51]. Nevertheless, when using the NOVA system to make dietary recommendations, it should be considered that some foods are difficult to classify [2]. Further research is needed to promote the application of this food classification system.

Considering the synergistic health-related effects of foods, it is of great importance to study dietary patterns instead of single foods or nutrients. UPFs consumption is increasing dominantly across the globe, especially in Western countries. It is consistent with the increased burden of NCDs attributable to unhealthy diets. The role of some specific UPFs has been assessed, such as processed meats and sweetened beverages, showing positive associations with NCDs [52, 53]. In line with our findings, previous studies reported an inverse association between higher diet quality and risk of all-cause, cardiovascular disease, and cancer mortality [54–56]. Adherence to healthier diet patterns, which are characterized by a high consumption of unprocessed or minimally processed foods, was promoted to prevent NCDs. Decrease of cardiovascular disease burden with a healthier food system was observed in two modelling studies [57, 58]. A review of systematic reviews found that grain products and tea were protective, while processed meats and soft drinks tended to increase the risk [59]. Existing meta-analyses demonstrated that an optimal intake of several food groups could decrease the risk of coronary heart disease, stroke and heart failure [60]. Overall, UPFs consumption should be limited in prevention of NCDs.

The mechanism is multi-faceted. First, UPFs consumption is usually accompanied with high intake of fats, calories, sugars and salt, and low intake of micronutrients and fibre. The poor quality of dietary nutrients leads to the development of NCDs [61]. Processing, especially heat treatments, food additives and food packaging, can generate carcinogenicity and genotoxicity [25]. UPFs consumption increases added sugar intake, which is associated with obesity and several other health outcomes [19, 21, 62, 63]. Moreover, higher intake of UPFs induces changes in gut microbiota, serum C-reactive protein levels and lipoprotein profiles [64–67]. Displacement of unprocessed or minimally processed foods might play a potential role in decreased diet quality [37]. However, it still remains unclear what plays a leading role in the association. A better understanding of what really matters and how various aspects contribute to the effects is highly needed.

Our study has strengths. To our knowledge, this is the most comprehensive systematic review of the topic to date. We carried out extensive literature research. The occurrence of selection bias was reduced greatly due to the prospective design of all the cohort studies. Large number of participants might compensate for the inadequate number of studies of each health outcome. All the eligible studies made adequate adjustments of the potential confounding factors. In general, we provide strong implications for dietary policies and guidelines.

Several limitations should also be acknowledged. First, most cohort studies recruited university graduates or volunteers as study objects, who tended to be more health-conscious and had a lower UPFs consumption than the general population. This probably resulted in an underestimation of the association of interest. Second, as occurrence of some health outcomes took a long time, such as carcinogenic processes, the median follow-up was relatively inadequate. Besides, complete detection of outcomes could not be guaranteed. Third, epidemiological studies could not exclude reverse causality and residual confoundings. For cross-sectional studies, probability existed that participants changed their dietary habits after the occurrence of diseases. It tended to cause an underestimation of the results. Fourth, some misclassification in the NOVA system could not be ruled out. Ways applied for dietary assessment were not specifically designed for NOVA classification and UPFs consumption yet. However, substantial differences between the highest and the lowest group might reduce the bias to a great extent. It is also worth mentioning that no quantitative meta-analysis was conducted, which we hoped to be overcome with further research. Given ethical issues of conducting randomized controlled trials of risk factors, more well-designed epidemiological studies are needed to confirm these findings.

There are significant public health implications in our study. To date, prevention and control of NCDs are becoming a growing concern. UPFs are beginning to be recognized as an emerging health risk. The positive association between UPFs consumption and adverse health outcomes provides insights into dietary policies and guidelines. Encouraging a decrease in UPFs consumption and an increase in the proportion of unprocessed or minimally processed foods are a direct way to resolve the issue. Food taxation and surveillance on food marketing still play a vital role. Dietary guidelines, in accordance with the shift of the global food system and health, are a necessity to slow the prevalence of NCDs.

## Conclusion

This study indicated a positive association between UPFs consumption and risk of several health outcomes. Our results encouraged a decrease in UPFs consumption and an increase in the proportion of unprocessed or minimally processed foods, such as fruits and vegetables. Considering diet-related risk factors, we provided insights into NCDs occurrence and prevention. Large-scale prospective designed studies are needed to confirm our findings and to better understand the relative effects of various aspects in UPFs.

## Supplementary information

**Additional file 1: Supplyment 1:** Supplementary Text 1. Review protocol.

**Additional file 2: Supplyment 2: Supplementary Table 1.** Quality of cohort studies according to the Newcastle-Ottawa Scale (NOS). **Supplementary Table 2.** Quality of cross-sectional studies according to the JBI Critical Appraisal Checklist.

## Data Availability

The datasets supporting the conclusions of this article are included within the article and its additional files.

## Abbreviations

UPFs: Ultra-processed foods
NCDs: Noncommunicable diseases
CIs: Confidence intervals
HRs: Hazard ratios
ORs: Odds ratios
RRs: Relative risks
