# Supplementary material for: Consumption of ultra-processed foods and health outcomes: a systematic review of epidemiological studies
Source: Nutr J. 2020 Aug 20;19:86. doi: 10.1186/s12937-020-00604-1 (PMC7441617; doi:10.1186/s12937-020-00604-1)
Supplement: Supplementary file 1 — Additional file 1: Supplyment 1: Supplementary Text 1. Review protocol. [file 12937_2020_604_MOESM1_ESM.docx]

**Supplementary** **Text 1. Review protocol**

Consumption of Ultra-processed Foods and Health Outcomes: A Systematic Review of Epidemiological Studies

**Methods of the review:**

This systematic review is completed according to the MOOSE (Meta-analysis Of Observational Studies in Epidemiology) Statement.

**Data sources:**

3 electronic databases (Pubmed, EMBASE, Web of Science).

**Search terms:**

| **Database** | **Search terms** |
| --- | --- |
| Pubmed* | (((((food) OR foods) OR product) OR products)) AND ((("ultraprocessed") OR "ultra processed") OR "ultra-processed") |
| EMBASE** | 'ultra-processed food' OR 'ultra processed food' OR 'ultra-processed foods' OR 'ultra-processed foodstuffs' OR 'ultraprocessed food' OR 'ultraprocessed foods' |
| Web of Science | TS=(food OR foods OR product OR products) AND TS=("ultra processed" OR "ultra-processed" OR "ultraprocessed") |

*We searched Pubmed according to self-designed strategy due to no MeSH term about ultra-processed foods was found.

**We searched EMBASE with the Emtree 'ultra-processed food'.

**Additional searching:**

Reference lists of selected articles and some key journals.

**Inclusion/exclusion criteria**

**Inclusion criteria:**

Studies were included if they were published in a peer-reviewed journal and reported an association between exposure to consumption of ultra-processed foods and any health outcomes. No limits on year of publication. Studies in language other than English were excluded.

Details were as follows:

1. Population: general population, children, adolescents or adults. Non-representative samples were also considered including graduates and volunteers

2. Age range for exposure: all ages

3. Intervention/Exposure: consumption of ultra-processed foods

4. Comparison: individuals consumed the highest versus the lowest of ultra-processed foods

5. Outcome: any health outcomes

6. Outcome Measurement: health outcomes diagnosed by a health professional, but self-reported health outcomes also accepted

7. Study designs of interest: epidemiological studies, including cohort studies and cross-sectional studies

8. Sampling size: 500 or more participants

9. Effect estimates: hazard ratios [HRs], odds ratios [ORs], or relative risks [RRs]) with 95% confidence intervals (CIs)

**Exclusion criteria:**

Articles initially excluded if they are duplicates or if the title clearly demonstrates that the consumption of ultra-processed foods and health outcomes of interest are not the focus of the article. Articles are then excluded based on the following:

1. The article does not explore an association between ultra-processed foods and health outcomes.

2. The article is a review, letter, editorial, correction, experimental study or meeting abstract.

**Data extraction sheet**

Identification of study:

1. first authors’ information

2. year of publication

Characteristics of study:

3. study design

4. study setting and population (period, location, sample size and follow-up)

5. exposure measures

6. outcome measures

7. methodological features

8. funding

9. effect estimates (HRs, ORs, and RRs) with 95%CIs
