# Supplementary material for: Consumption of ultra-processed foods and health outcomes: a systematic review of epidemiological studies
Source: Nutr J. 2020 Aug 20;19:86. doi: 10.1186/s12937-020-00604-1 (PMC7441617; doi:10.1186/s12937-020-00604-1)
Supplement: Supplementary file 2 — Additional file 2: Supplyment 2: Supplementary Table 1. Quality of cohort studies according to the Newcastle-Ottawa Scale (NOS). Supplementary Table 2. Quality of cross-sectional studies according to the JBI Critical Appraisal Checklist. [file 12937_2020_604_MOESM2_ESM.docx]

**Supplementary Table 1. Quality of cohort studies according to the Newcastle-Ottawa Scale (NOS)**

| Studies | Selection | | | | Comparability | Outcome | | | Quality score (Number of stars) |
| --- | --- | --- | --- | --- | --- | --- | --- | --- | --- |
|  | **Representativeness of the exposed cohort** | **Selection of the non exposed cohort** | **Ascertainment of exposure** | **Demonstration that outcome of interest was not present at start of study** | **Comparability of cohorts on the basis of the design or analysis** | **Assessment of outcome** | **Was follow up long enough for outcomes to occur** | **Adequacy of follow up of cohorts** |  |
| Rico-Campà,  2019 | ***** | ***** | ***** | ***** | ****** | ***** | ***** | ***** | **9** |
| Schnabel,  2018 | **-** | ***** | ***** | ***** | ****** | ***** | ***** | **-** | **7** |
| Blanco-Rojo,  2019 | ***** | ***** | ***** | ***** | ****** | ***** | ***** | ***** | **9** |
| Kim, 2019 | ***** | ***** | ***** | ***** | ****** | ***** | ***** | **-** | **8** |
| Srour,  2019 | **-** | ***** | ***** | ***** | ****** | ***** | ***** | **-** | **7** |
| Mendonça,  2017 | **-** | ***** | ***** | ***** | ****** | ***** | ***** | ***** | **8** |
| Schnabel,  2018 | **-** | ***** | ***** | ***** | ****** | ***** | ***** | **-** | **7** |
| Mendonça,  2016 | **-** | ***** | ***** | ***** | ****** | ***** | ***** | ***** | **8** |
| Adjibade,  2019 | **-** | ***** | ***** | ***** | ****** | ***** | ***** | ***** | **8** |
| Gómez‑  Donoso,  2019 | **-** | ***** | ***** | ***** | ****** | ***** | ***** | ***** | **8** |
| Fiolet,  2018 | **-** | ***** | ***** | ***** | ****** | ***** | **-** | **-** | **6** |
| Sandoval-Insausti,  2019 | ***** | ***** | ***** | ***** | ****** | ***** | **-** | ***** | **8** |

**Supplementary Table 2. Quality of cross-sectional studies according to the JBI Critical Appraisal Checklist**

| Studies | 1. Were the criteria for inclusion in the sample clearly defined? | 2. Were the study subjects and the setting described in detail? | 3. Was the exposure measured in a valid and reliable way? | 4. Were objective, standard criteria used for measurement of the condition? | 5.Were confounding factors identified? | 6. Were strategies to deal with confounding factors stated? | 7. Were the outcomes measured in a valid and reliable way? | 8. Was appropriate statistical analysis used? |
| --- | --- | --- | --- | --- | --- | --- | --- | --- |
| Steele,2019 | Yes | Yes | Yes | Unclear | Yes | Yes | Yes | Yes |
| Lavigne-Robichaud,2018 | Yes | Yes | Yes | Unclear | Yes | Yes | Yes | No |
| Juul,2018 | Yes | Yes | Yes | Yes | Yes | Yes | Yes | Yes |
| Louzada,2015 | Yes | Yes | Unclear | Yes | Yes | Yes | Yes | Yes |
| Nardocci,2019 | Yes | Yes | Yes | Yes | Yes | Yes | No | Yes |
| Silva,2018 | Yes | Yes | Yes | Yes | Yes | Yes | Yes | No |
| Sartorelli,2019 | Yes | Yes | Unclear | Yes | Yes | Yes | Yes | No |
| Melo,2018 | Unclear | Yes | Yes | Unclear | Yes | Yes | No | Yes |
